# The efficacy and safety of tranexamic acid for reducing blood loss following simultaneous bilateral total knee arthroplasty: a multicenter retrospective study

**DOI:** 10.1186/s12891-019-2692-z

**Published:** 2019-07-12

**Authors:** Guorui Cao, Guo Chen, Qiang Huang, Zeyu Huang, Peter G. Alexander, Hang Lin, Hong Xu, Zongke Zhou, Fuxing Pei

**Affiliations:** 1 Department of Orthopaedic surgery, West China Medical School, West China Hospital, Sichuan University, 37# Guoxue Road, Chengdu, Sichuan Province 610041 People’s Republic of China; 2 Department of Orthopedics, The Eighth Affiliated Hospital, Sun Yat-sen University, Shenzhen, Guangdong Province People’s Republic of China; 3 Center for Cellular and Molecular Engineering, Department of Orthopaedic Surgery, University of Pittsburgh School of Medicine, Pittsburgh, USA

**Keywords:** Total knee arthroplasty, Simultaneous bilateral, Tranexamic acid, Blood loss, Transfusion

## Abstract

**Background:**

The purpose of the study was to evaluate whether tranexamic acid (TXA) administration could reduce blood loss and transfusion risk after simultaneous bilateral total knee arthroplasty (SBTKA).

**Methods:**

As a multicenter retrospective study, a total of 575 patients were assigned into three groups on the basis of TXA usage, including intravenous (IV) group (1 g IV TXA 5–10 min prior to the incision), combined group (1 g IV TXA combined with intra-articular injection of 1 g TXA prior to the closure every knee) and control group (no TXA use). The primary outcomes were total blood loss (TBL). The secondary outcomes were maximum hemoglobin (Hb) and hematocrit (Hct) drop, transfusion rate, drain volume, length of stay, hospitalization expenses and the incidence of complications.

**Results:**

The mean TBL in control group (1685.0 ± 571.4 mL) were higher than that in IV group (1061.1 ± 689.6 mL, *p* = 0.006 and combined group (988.3 ± 559.3 mL, *p* = 0.003). The maximum Hb and Hct drop in combined group (28.5 ± 13.4 g/L, *p* = 0.016; 0.074 ± 0.053, *p* < 0.001) and IV group (28.8 ± 14.5 g/L, *p* = 0.025; 0.082 ± 0.056, *p* = 0.001) were lower than those in control group (33.4 ± 14.0; 0.131 ± 0.049). But the difference between IV and combined groups was not significant. The similar trend was detected on drain volume, length of stay and hospitalization expenses. The incidence of complications did not differ significantly among the three groups (*p* > 0.05).

**Conclusions:**

The study indicates that TXA could reduce blood loss with no apparent increase in the incidence of complications during SBTKA.

## Background

Total knee arthroplasty (TKA) is always accompanied by massive blood loss, which increase the risk of anemia and allogeneic blood transfusion, leading to higher incidence of wound complications, immunologic reactions, transmission of disease and delaying functional recovery [1–3]. Therefore, blood management associated with TKA is an important and widely-discussed topic for surgeons. Blood management for simultaneous bilateral total knee arthroplasty (SBTKA) patients is more challenging than in primary TKA.

The TKA could motivate the process of fibrinolysis, which is the main reason of perioperative blood loss [4]. It was reported that Tranexamic acid (TXA) was effective and safe in inhibiting hyperfibrinolysis and reducing blood loss in SBTKA [5–7]. TXA could be applied intravenously, topically or combination of multiple routes. Previous studies have showed that intravenous (IV) or local administration of TXA are effective and safe in reducing blood loss and transfusion need in SBTKA [5, 6, 8]. However, studies to evaluate the efficacy of combined IV and intra-articular administration of TXA are rare. Moreover, the study sample of previous studies was too small to provide powerful evidence for efficacy and safety of TXA following SBTKA. Thus, the purpose of the study was to evaluate whether TXA administration could reduce blood loss and transfusion risk after SBTKA through a multicenter, large sample, retrospective study.

## Methods

### Patients and design

A multicenter database was established to evaluate the efficacy and safety of perioperative management for total hip and knee arthroplasty in China in January 2013, sponsored by the Chinese Health Ministry (201302007). This study was a multicenter retrospective study and the data were from 4 teaching hospitals in the database [9]. The study was approved by our institutional review board (2012–268).

We identified all cases of SBTKA using International Classification of Diseases, Tenth Revision, Clinical Modification (ICD-10-CM) procedure codes from January 2015 to June 2018. SBTKA was defined as procedure performed under a single episode of anesthesia. The patients with a primary diagnosis code of infection, fracture, or malignancy, with incomplete demographic information, primary and bilateral single-stage procedures were excluded. In addition, we excluded those presence of a congenital or acquired clotting disorder, a history of deep venous thrombosis (DVT) or pulmonary embolism (PE), cardiovascular problems, or a known allergy to TXA. What was more, the patients were excluded when the intraoperative blood loss was more than 400 mL during the first knee surgery. Totally, 575 patients undergoing SBTKA were recruited. The patients were divided into intravenous (IV) group (1 g/100 mL IV TXA 5–10 min before the skin incision), combined (IV + topical use) group (Intra-articular injection of 1 g/100 mL before the closure every knee, totally 2 g per patient) and control group (no TXA use).

#### Surgery procedure and perioperative management

All the TKAs were performed using the midline skin incision and medial parapatellar approach. A measured resection technique was used in the surgery. The tourniquet was utilized in all patients which was inflated prior to skin incision and deflated immediately prior to closure. All patients received drains, which were removed when the volume of drain was less than 30 mL/h. The procedures were conducted under general or lumbar anesthesia. Patients received half-dose of low-molecular-weight heparin (LMWH; 2000 IU in 0.2 ml; Clexane, Sanofi-Aventis, France) or 10 mg Rivaroxaban (Xarelto, Bayer, Germany) 6–8 h postoperatively, repeating once a day for 14 days. Doppler ultrasound examinations were used routinely to detect DVT. Transfusions were applied if the hemoglobin (Hb) level was < 70 g/L or 70–100 g/L with symptoms of anemia, such as bad mental status, palpitation or shortness of breath not due to other causes.

### Outcome measurements

The primary outcomes were total blood loss (TBL), calculated on the basis of the Gross and Nadler formula [10, 11]. TBL = patient’s blood volume (PBV) × (Hct_pre_-Hct_post_)/Hct_ave_ (Hct_pre_ = the initial preoperative Hct level, Hct_post_ = the Hct on the morning of POD3. PBV = k_1_ × height (m)^3^ + k_2_ × weight (kg) + k_3_ (k_1_ = 0.3669, k_2_ = 0.03219, and k_3_ = 0.6041 for men; and k_1_ = 0.3561, k_2_ = 0.03308, and k_3_ = 0.1833 for women, Hct_ave_ = the average of the Hct_pre_ and Hct_post_). If either reinfusion or allogeneic transfusion was performed, the TBL was equal to the loss calculated from the change in Hct plus the volume transfuse [12]. The secondary outcomes were maximum Hb and hematocrit (Hct) drop, transfusion rate, drain volume, length of stay (LOS), hospitalization expenses and the incidence of complications. The blood sample was collected and analyzed preoperatively, postoperative day (POD) 1 and 3. The maximum Hb and Hct drop was defined as the difference between the preoperative Hb, Hct and the lowest postoperative Hb, Hct during hospitalization.

### Statistical analysis

The SPSS version 22.0 (SPSS Inc. USA) was used to evaluate all statistical analyses. The continuous variables were compared using the one-way analysis of variance, Wilcoxon Mann-Whitney U test or independent t-test. The categorical variables were compared using Pearson chi-square test or Fisher exact test. The *p*-value < 0.05 was considered to be statistically significant.

## Results

### Patients’ demographics

A total of 623 patients recruited for SBTKA were screened for eligibility from January 2015 to June 2018. However, 48 patients were excluded because of the exclusion criteria and remaining 575 patients (192 in IV group, 135 in combined group and 248 in control group) were observed and studied. The baseline characteristics of the patients among the three groups were comparable (Table 1).

**Table 1:** Baseline characteristics

Baseline Characteristic	IV group (*n* = 192)	Combined group (*n* = 135)	Control group (*n* = 248)	*P*
Age (y)	66.2 ± 7.9	65.8 ± 9.1	66.6 ± 9.5	0.675
Gender(M/F)	32/160	23/112	43/205	0.983
BMI (kg/ *m*^2^)	27.1 ± 3.8	27.6 ± 3.3	27.0 ± 3.5	0.230
Diagnose (OA/IA)	183/9	126/9	238/10	0.514
Hypertension	67 (34.9%)	59 (43.7%)	85 (34.3%)	0.154
Diabetes	20 (10.4%)	17 (12.6%)	21 (8.5%)	0.433
Preoperative Hb (g/L)	140.8 ± 12.2	143.2 ± 10.8	141.4 ± 10.4	0.432
Preoperative Hct	0.415 ± 0.035	0.420 ± 0.032	0.415 ± 0.037	0.758
PBV (mL)	4047.3 ± 505.6	4060.4 ± 559.1	4020.3 ± 532.1	0.749
Anticoagulation methods				0.398
LMWH	109	86	153	
Rivaroxaban	83	49	95	
Anesthesia method				0.578
General	152	112	195	
Regional	40	23	53	
ASA class				0.852
1–2	188	131	241	
≥3	4	4	7	
Operating time (min)	120.3 ± 37.1	153.8 ± 43.9	139.3 ± 39.1	< 0.001*

*BMI* Body mass index = Weight/ Height^2^, *OA* Osteoarthritis, *IA* Inflammatory arthritis, *Hb* Hemoglobin, *Hct* Hematocrit, *PBV* patient blood volume, *LMWH* low-molecular-weight heparin, *ASA* American Society of Anesthesiologists

*Significant difference

### Blood loss

The mean TBL in control group (1685.0 ± 571.4 mL) were higher than that in IV group (1061.1 ± 689.6 mL, *p* = 0.006) and combined group (988.3 ± 559.3 mL, *p* = 0.003). The transfusion rate in control group (23.0%) were higher than that in IV group (14.1%, *p* = 0.018) and combined group (11.1%, *p* = 0.005). The difference of TBL and transfusion rate between IV and combined groups was not significant. The similar trend was detected on drain volume. The maximum Hb and Hct drop in combined group (28.5 ± 13.4 g/L, *p* = 0.016; 0.074 ± 0.053, *p* < 0.001) and IV group (28.8 ± 14.5 g/L, *p* = 0.025; 0.082 ± 0.056, *p* = 0.001) were lower than those in control group (33.4 ± 14.0; 0.131 ± 0.049). These statistical difference existed in LOS and hospitalization expenses between IV group and control group, as well as combined group and control group. However, the differences between IV and combine groups did not reach statistical significance. The outcome of blood loss was summarized in Table 2.

**Table 2:** Comparison of Blood Loss, LOS and expenses

Variable	IV group (*n* = 192)	Combined group (*n* = 135)	Control group (*n* = 248)	*P*	*P*1	*P*2	*P*3
TBL (mL)	1061.1 ± 689.6	988.3 ± 559.3	1685.0 ± 571.4	0.006*	0.693	0.003*	0.003*
Transfusion rate	27 (14.1%)	15 (11.1%)	57 (23.0%)	0.005*	0.818	0.018*	0.005*
Drain volume	432.9 ± 262.6	393.2 ± 250.3	663.9 ± 199.4	< 0.001*	0.348	0.005*	< 0.001*
Maximum Hb drop	28.8 ± 14.5	28.5 ± 13.4	33.4 ± 14.0	0.015*	0.710	0.025*	0.016*
Maximum Hct drop	0.082 ± 0.056	0.074 ± 0.053	0.131 ± 0.049	0.001*	0.614	0.001*	< 0.001*
LOS	7.8 ± 6.6	7.7 ± 4.8	10.1 ± 3.8	0.781	< 0.001*	< 0.001*	< 0.001*
Expenses ∆	93,625.8 ± 16,516.4	94,255.1 ± 10,453.5	99,174.1 ± 14,032.7	0.005*	0.676	0.005*	0.006*

*TBL* Total blood loss, *Hb* Hemoglobin, *Hct* Hematocrit, *POD* postoperative day. LOS: length of stay; P represents P value of IV vs combined vs control group, P1 represents P value of IV vs combined group, P2 represents P value of IV vs control group, P3 represents *P* value of combined vs control group

∆ Results were presented as Chinese yuan

*Significant difference

### Complications

There were 1 patient in IV group, 1 in combine group and 3 in control group developing DVT in the study. In addition, two patients in IV group and one patient in control group developed superficial infection. No PE, cardiac infarction, acute renal failure or deep infection were detected. The incidence of complications was similar among the three groups (Table 3). All patients were discharged uneventfully after symptomatic treatment.

**Table 3:** Complications

Complications	IV group (*n* = 192)	Combined group (*n* = 135)	Control group (*n* = 248)	*P*
PE	0	0	0	–
DVT	1	1	3	0.730
Cardiac infarction	0	0	0	–
Acute renal failure	0	0	0	–
Superficial infection	2	0	1	0.412
Deep infection	0	0	0	–

*PE* pulmonary embolism, *DVT* deep venous thrombosis, *CMVT* calf muscular vein thrombosis

## Discussion

Perioperative blood loss in SBTKA has been a crucial issue because SBTKA is associated much blood loss and higher risk of bleeding complications [13, 14]. The efficacy and safety of TXA to reduce blood loss has been well studied recently. However, the usage was single and the combination of TXA was rare [5, 6, 8]. Moreover, the research was small. There was a paucity of multicenter, large sample studies on the efficacy and safety of TXA in the setting of SBTKA. In this research, we found that IV TXA or combining IV and intra-articular injection of TXA both could decrease blood loss, drain volume, Hb and Hct drop, as well as LOS and expenses following SBTKA.

The application of TXA has been shown to reduce blood loss and transfusion requirements in patients undergoing primary TKA. However, the studies related to SBTKA using TXA are sparse [5, 6, 8, 15, 16]. A randomized controlled trail conducted by Chen et al. showed that fixed dose of IV TXA (1 g/100 mL) for patients undergoing SBTKA was effective and safe in reducing total blood loss and allogeneic blood transfusion needs without any additional thromboembolic risk [6]. Karam and colleague’s study came to a similar conclusion [16]. Bagsby et al. conducted that IV TXA (10 mg/kg, preoperatively) was an effective tool in reducing the transfusion rates in SBTKA [5]. Karaaslan et al. evaluated the usage of combining IV (15 mg/kg preoperatively and continuing 10 mg/kg/h for 3 h postoperatively) and intra-articular TXA (3 g/100 mL saline after clamping of the drain) administration, showing that TXA could reduce blood loss with negligible side effects during SBTKA [15]. Another study performed by Suh et al. found that IV iron supplementation combined with intra-articular administration of TXA (2 g/20 ml saline) was an effective strategy for reducing Hb drop and transfusion risk following SBTKA [8]. Prieto et al. found that IV TXA (20 mg/kg) in SBTKA could effectively reduce blood loss, maintain postoperative Hb and Hct levels, and significantly decrease blood transfusion rates [7]. However, the number of cases was only 20–60 and persuasiveness was a little low. So we conducted this retrospective, large sample research, showing that TXA could reduce blood loss, transfusion rate, drain volume, maximum Hb and Hct drop, LOS and hospitalization expenses. But the difference of administration of single IV TXA and combination of IV and topical TXA was not significant.

Comparing with the expenses in control group, the expenses in IV and combined group decreased significantly. The reason might be the decreasing of LOS and allogenetic transfusion needs. It was reported that multiple doses of TXA could further reduce blood loss, transfusion rate and LOS, as well as oral TXA had greater cost-effectiveness in primary TKA [17, 18]. So the expenses of patients undergoing SBTKA could be further reduced.

To our best knowledge, there have been no studies to specifically evaluate the difference between single IV TXA and combined TXA (IV and topical TXA) in the setting of SBTKA. In this study, we found that the efficacy of single IV TXA and combination of IV as well as topical (intra-articular injection) TXA was similar, indicating combined application of TXA failed to get better results in SBTKA. However, the study of Xiong et al. showed that the combined group (IV and topical TXA) had significantly less blood loss than the IV group following primary TKA [19], which was not consistent with our study. First of all, the surgery was different, SBTKA was associated with much blood loss compared with primary TKA. In addition, the subgroup analyses in Xiong’s study showed that the combined group was less total blood loss in topical TXA dose > 1.5 g following primary TKA. However, the dose of topical TXA was only 2 g in the setting of SBTKA, approximately equal to1g each TKA in our study, which was insufficient. Therefore, we just found the downtrend in combined group in blood loss but the difference between IV and combined groups was not significant after SBTKA.

As an anticoagulant, the safety of TXA had been concerned and evaluated widely. Table 4 summarized the incidence of complications from previous and this studies for patients undergoing SBTKA [5–8, 15, 16, 20]. The incidence of thromboembolic events was very low and was similar between patients with or without TXA.

**Table 4:** The baseline characteristics of patients of previous studies

Location of Study	Usage	DVT	PE	Other adverse events	Statistical differences
UAE (2011)	IV	Not mentioned	1/20	Not mentioned	NS
U.S. (2013)	IV	0	0	0	NS
Turkey (2014)	IV and Topical	1/41	0	0	NS
U.S. (2015)	IV	2/46	0	9/46	NS
China (2016)	IV	0	0	0	NS
South Korea (2017)	Topical	Not mentioned	Not mentioned	Not mentioned	NS
U.S. (2017)	IV	0	0	0	NS
China (2019)	IV and topical	1/192 and 1/135	0	2/192 and 0/135	NS

*DVT* deep venous thrombosis, *PE* pulmonary embolism, *UAE* United Arab Emirates, *IV* intravenous, *NS* not significant

There are some surgery-associated factors which are associated with preoperative blood loss during TKA, especially the tourniquet and navigation. A North American database analysis showed the use of navigation in TKA could result in less perioperative transfusions [21]. In addition, our previous study indicated that the application of tourniquet could increase the transfusion risk in SBTKA [9]. However, we did not use the navigation and the tourniquet was applied for all patients in this study considering much blood loss in SBTKA. We have recognized the advantage of navigation and disadvantage of tourniquet. We will consider performing navigation TKA without tourniquet in the near future.

The study was carefully designed to reduce bias, but several limitations still existed. Firstly, the blood sample were mainly measured on POD1 as well as POD3 and the TBL was calculated based on level of Hct on POD3 in most cases. The Hct may continue to decline so the TBL that we calculated was not exact TBL. However, we believed that the potential inaccuracy due to Hct did not impact the result because of the consistency of calculation method among the three groups. Second, the perioperative management methods were not completely consistent among the different hospitals and experience of surgeons varied. In addition, the follow-up time was merely 1 month, which could be short of power to sufficiently evaluate the risk of DVT and other complications. Therefore, further studies with long-term follow-up are requisite.

## Conclusion

The study indicates that TXA could reduce blood loss with no apparent increase in the incidence of complications during SBTKA.

## Data Availability

The datasets used and/or analyzed during the current study are available from the corresponding author on reasonable request.

## Abbreviations

DVT: Deep venous thrombosis
Hb: Hemoglobin
Hct: Hematocrit
IV: Intravenous
LMWH: Low-molecular-weight heparin
LOH: Length of stay
PBV: Patient’s blood volume
PE: Pulmonary embolism
POD: Postoperative day
SBTKA: Simultaneous bilateral total knee arthroplasty
TBL: Total blood loss
TKA: Total knee arthroplasty
TXA: Tranexamic acid
